# Is serum cholinesterase level a predictor of the extent of organ involvement in immunoglobulin G4-related disease?

**DOI:** 10.1093/rap/rkaa031

**Published:** 2020-07-07

**Authors:** Kazu Hamada-Ode, Mitsuharu Yoshida, Yoshio Terada, Yoshinori Taniguchi

**Affiliations:** ^1^ Department of Endocrinology, Metabolism, Nephrology and Rheumatology, Kochi Medical School Hospital, Nankoku, Japan

**Key message**

- Serum cholinesterase could be new biomarker and predict the extent of organ involvement in IgG4-related disease.

Dear Editor, IgG4-related disease (IgG4-RD) is a fibroinflammatory condition generally characterized by tumefactive lesions and elevated serum IgG4 concentration [1]. Typical pathological findings include dense tissue fibrosis with a storiform pattern, a diffuse lymphoplasmacytic infiltrate with an abundance of IgG4-positive plasma cells, mild to moderate eosinophilia and obliterative phlebitis. Serologically, the most important feature is an elevated serum IgG4 concentration; however, serum IgG4 concentrations are normal in some patients with IgG4-related periaortitis/retroperitoneal fibrosis or type 1 autoimmune pancreatitis. In contrast, serum IgG4 is usually high when the kidney is involved [2]. Reflecting an allergic predisposition, many patients have serum IgE elevation and/or eosinophilia [3]. Hypergammaglobulinaemia is also a distinctive feature of IgG4-RD [4]. Another very important serological feature is hypocomplementaemia [4], and Saeki *et al*. [5] reported that serum complement level might be a useful biomarker to monitor relapse in IgG4-related tubulointerstitial nephritis. However, the mechanism of IgG4-RD remains unclear. We often encounter cases of IgG4-RD with low levels of serum cholinesterase (ChE). Therefore, we aimed to clarify the clinical significance of serum ChE in IgG4-RD patients. The age, sex, laboratory findings and number of organs involved in 30 Japanese patients with IgG4-RD [mean (s.d.) age, 67 (9) years; 21 men, 9 women] were assessed retrospectively and also compared with healthy controls (Table 1). This study was approved by the Ethics Committee of Kochi Medical School Hospital and conducted in accordance with the Declaration of Helsinki. All clinical information was obtained after the patients had given their informed consent.

**Table 1:** Main characteristics of patients with IgG4-related disease and healthy controls

Characteristic	Healthy controls (*n* = 10)	Total IgG4-related disease (*n* = 30)	IgG4-RD with normal cholinesterase (*n* = 16)	IgG4- related disease with low c holinesterase (*n* = 14)
Age, mean (s.d.), years	60 (7)	67 (9)	65 (6)	70 (11)
Male : female	7 : 3	21 : 9	10 : 6	9 : 5
IgG, mean (s.d.), mg/dl	NA	3105 (1716)	2374 (1207)	3744 (908)
IgG4, mean (s.d.), mg/dl	NA	722.8 (592.4)	538.3 (600.0)	866.2 (578.8)
CRP, mean (s.d.), mg/dl	NA	0.5 (0.4)	0.3 (0.2)	0.6 (0.4)
50% hemolytic complement activity, mean (s.d.), U/ml	NA	30.5 (25.1)	46.4 (16.8)	18.2 (24.2)^a^
Complement 3, mean (s.d.), mg/dl	NA	86.7 (49.2)	117.8 (5.0)	73.6 (55.8)
Complement 4, mean (s.d.) mg/dl	NA	18.7 (13.5)	25.4 (13.3)	15.1 (12.7)
Cholinesterase, mean (s.d.), U/l	326.6 (56.0)	236.6 (112.7)	328.2 (61.7)	131.9 (42.7)^a^^,^^b^
Number of organs involved, mean (s.d.)	NA	3.1 (1.7)	1.9 (1.3)	4.3 (1.3)^a^

a *P * < * *0.05 *vs* IgG4-related disease with normal cholinesterase.

b *P * < * *0.05 *vs* healthy controls.

NA: not available.

The mean serum IgG and IgG4 levels were 3105 (1716) and 722.8 (592.4) mg/dl, respectively. Fourteen IgG4-RD patients had significantly lower levels of serum ChE [mean 131.9 (42.7) U/l] than those of healthy controls (*P *<* *0.05) and 16 IgG4-RD patients had normal levels [mean 328.2 (61.7) U/l; *P *<* *0.05]. Notably, serum ChE levels in most patients were elevated 1 month after CS therapy (Supplementary Fig. S1, available at *Rheumatology Advances in Practice* online) and decreased again during flare-up in some cases (data not shown).

Moreover, the number of organs involved in IgG4-RD cases with lower levels of serum ChE [mean (s.d.), 4.3 (1.3)] was significantly greater than that in IgG4-RD cases with normal levels of serum ChE [1.9 (1.3); *P *<* *0.05; Table 1]. In all IgG4-RD cases, ChE levels wereinversely correlated with the number of organs involved (data not shown). Next, we divided total IgG4-RD cases to two groups [multiple organs involved (more than three) and limited organ involvement (fewer than 2)] and analysed the relationship with serum ChE. Serum ChE levels were significantly lower in IgG4-RD cases with renal involvement than in those without renal involvement (*P* = 0.01; Supplementary Fig. S2A, available at *Rheumatology Advances in Practice* online) and significantly lower in the multiple organ involvement group (*n* = 12) than in the limited organ involvement group (*n* = 18) (*P *=* *0.001; Supplementary Fig. S2A, available at *Rheumatology Advances in Practice* online). Stepwise analysis of serum ChE activity, the number of organs involved and several serological markers demonstrated that serum ChE activity was well correlated with the number of organs involved (β * *=  0.248, *F *=* *8.941; Supplementary Fig. S2B, available at *Rheumatology Advances in Practice* online).

Finally, some laboratory data of IgG4-RD cases with multiple organ involvement, ANCA-associated vasculitis (AAV; *n* = 10) and SS; *n* = 10) were examined comparatively because of exclusion of effects by hepatic protein synthetic ability and inflammation. Serum IgG and albumin levels were significantly lower in AAV cases than in IgG4-RD cases with multiple organ involvement and SS (Supplementary Fig. S3A and C, available at *Rheumatology Advances in Practice* online). CRP concentrations were significantly higher in AAV cases than in IgG4-RD cases with multiple organ involvement and SS (Supplementary Fig. S3B, available at *Rheumatology Advances in Practice* online). Despite these results, serum ChE levels were significantly lower in IgG4-RD cases with multiple organ involvement than in AAV and SS cases (Supplementary Fig. S3D, available at *Rheumatology Advances in Practice* online). The markers indicating hepatic function, such as alanine aminotransferase, γ-glutamyl transpeptidase and platelets, revealed no significant differences between IgG4-RD cases with multiple organ involvement, AAV cases and SS cases (data not shown). Although we could not elucidate the mechanism of the change in serum ChE, serum ChE is a potential novel predictor of the extension of the IgG4-RD lesions.

In fact, recently, several biomarkers, including IgG2, serum soluble IL-2 receptor and CC chemokine ligand 18, have been shown to indicate inflammation and fibrosis and to diagnose and predict treatment response accurately in IgG4-RD [6]. However, simpler tests are required to evaluate the extension of organ involvement in IgG4-related diseases. Therefore, we focused on the serum ChE level, which is one of the common blood tests, as a biomarker in IgG4-RD.

Serum ChE comprises acetylcholinesterase and butyrylcholinesterase (BChE). In Japan, the measurement of serum ChE generally means that of BChE. The tissues with the highest BChE activity were found to be the liver, lungs, spleen, kidney, pancreas, stomach, small intestine, cerebellum, heart and plasma [7]. The tissues with the highest BChE activity are common sites of IgG4-RD lesions. Therefore, although this is a hypothesis, multiple organ and tissue involvement in IgG4-RD might decrease BChE activity in organs and tissue levels. Further investingation regarding ChE is required to determine its clinical importance in IgG4-RD patients.

In conclusion, the serum ChE level might be a new biomarker and clinically useful predictor indicating the extension of organ involvement in IgG4-RD. Further studies are necessary to gain a clear understanding of the relationship between the serum ChE level and IgG4-RD.

## Supplementary Material

rkaa031_Supplementary_Data
