# Supplementary material for: Is serum cholinesterase level a predictor of the extent of organ involvement in immunoglobulin G4-related disease?
Source: Rheumatol Adv Pract. 2020 Jul 7;4(2):rkaa031. doi: 10.1093/rap/rkaa031 (PMC7494081; doi:10.1093/rap/rkaa031)
Supplement: rkaa031_Supplementary_Data [file rkaa031_supplementary_data.zip › SUPPLEMENTARY MATERIAL RAP 1.docx]

**SUPPLEMENTARY MATERIAL**

**Supplementary Figure legends**

**Supplementary Figure S1.** **Serum ChE changes before and after steroid treatments.**

**Supplementary Figure S2.** **The correlation between serum ChE and other parameters**. **(A)** The relationship between serum ChE levels, renal involvements, and multiple or limited organ involvements. **(B)** Stepwise analysis between serum ChE activity, number of organ involvements and several serological markers.

**Supplementary Figure S3.** **Comparison of several parameters between IgG4-RD with multiple lesions, ANCA-associated vasculitis and Sjogren syndrome.** **(A)** Comparison of serum IgG levels. **(B)** Comparison of serum CRP levels. **(C)** Comparison of serum Alb levels. **(D)** Comparison of serum ChE levels.
