# Supplementary material for: Is serum cholinesterase level a predictor of the extent of organ involvement in immunoglobulin G4-related disease?
Source: Rheumatol Adv Pract. 2020 Jul 7;4(2):rkaa031. doi: 10.1093/rap/rkaa031 (PMC7494081; doi:10.1093/rap/rkaa031)
Supplement: rkaa031_Supplementary_Data [file rkaa031_supplementary_data.zip › Revised Supplementary Figure.pptx]

## Slide 1
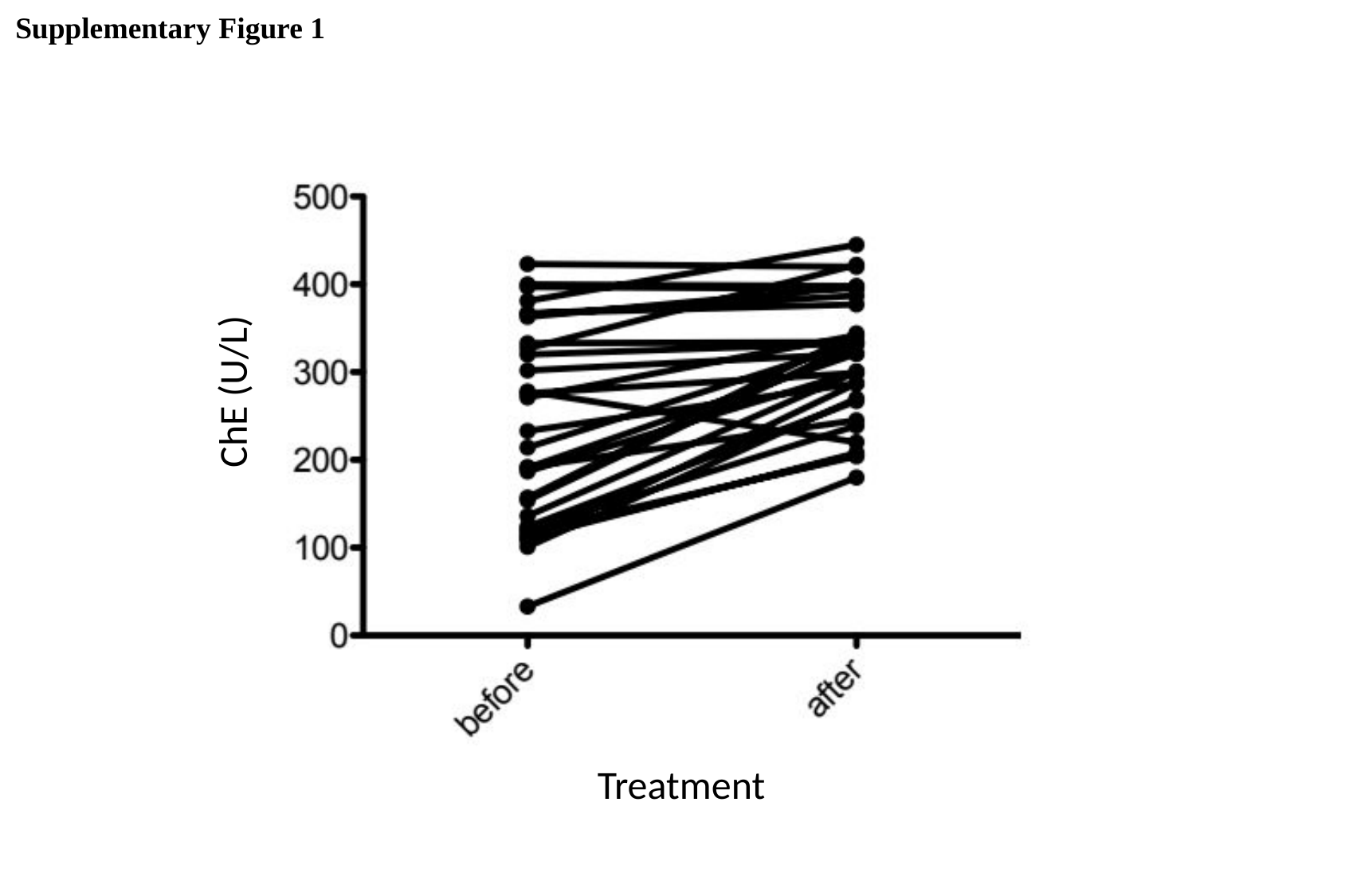

Supplementary Figure 1
ChE (U/L)
Treatment

## Slide 2
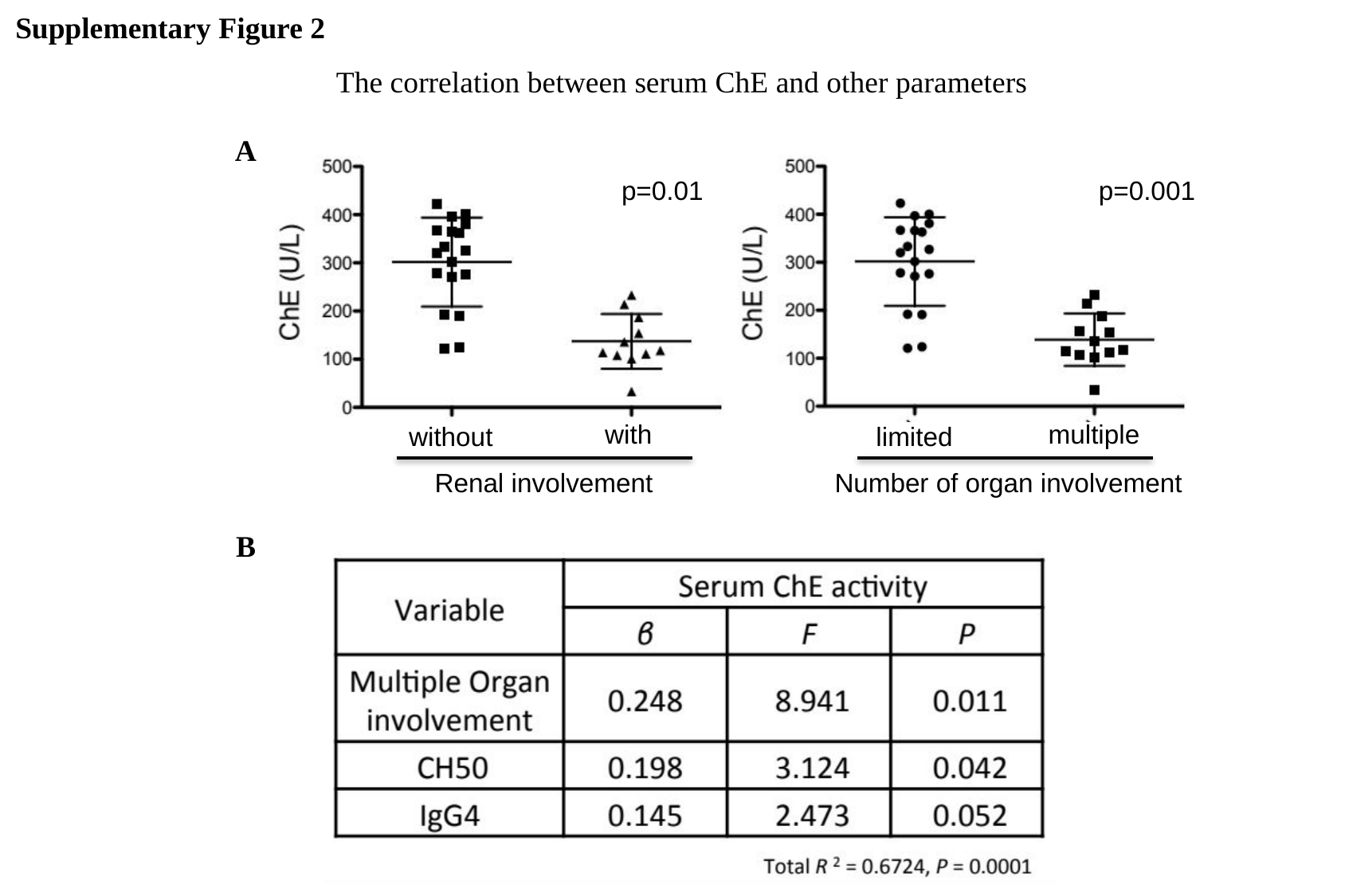

Supplementary Figure 2
The correlation between serum ChE and other parameters
A
p=0.01
p=0.001
with
multiple
without
limited
Renal involvement
Number of organ involvement
B

## Slide 3
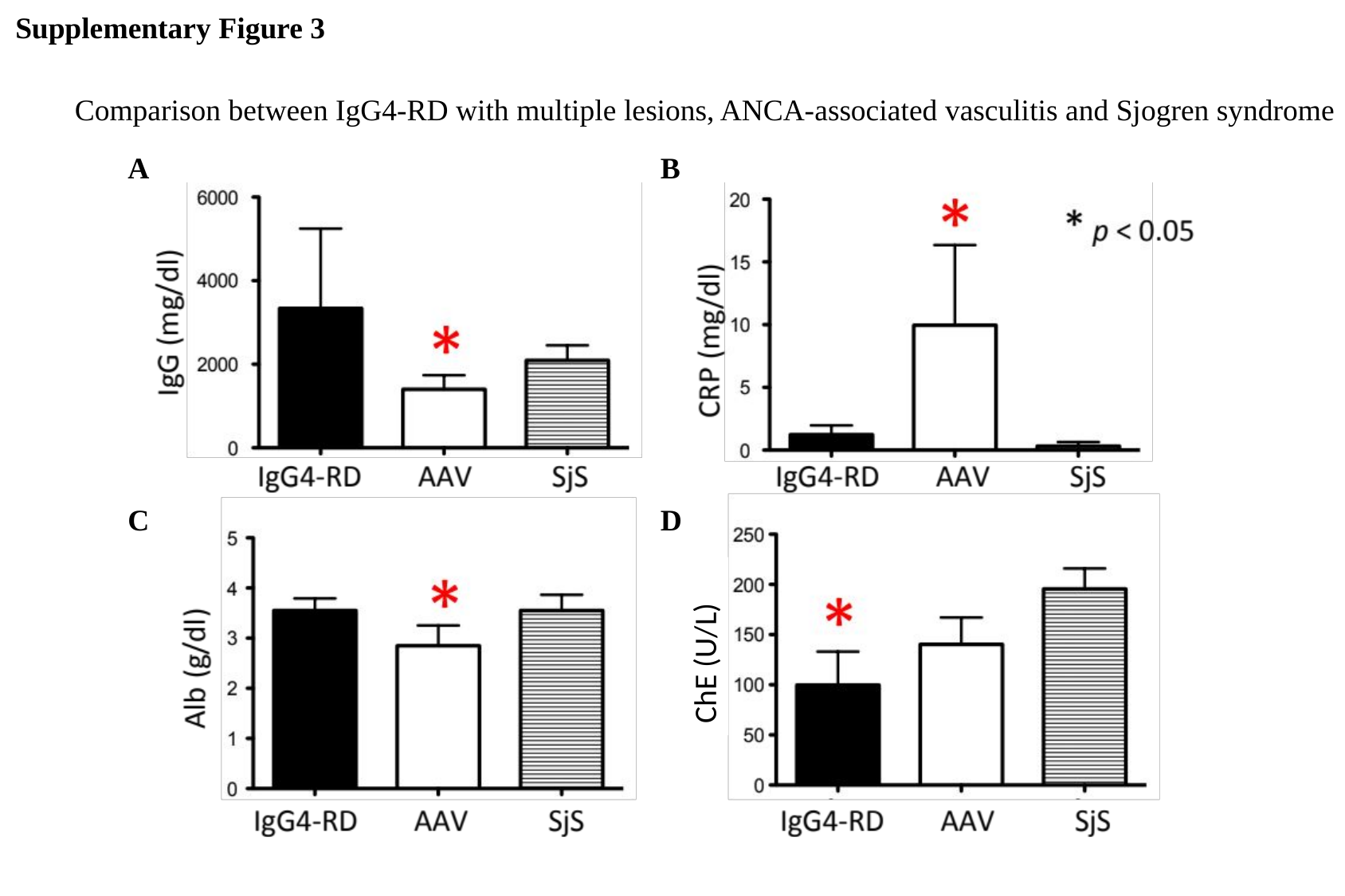

Supplementary Figure 3
Comparison between IgG4-RD with multiple lesions, ANCA-associated vasculitis and Sjogren syndrome
A
B
C
D
ChE (U/L)
